# Multimodal MRI reveals distinct hippocampal subregional alterations in *de novo* Parkinson’s disease across the cognitive spectrum

**DOI:** 10.3389/fnagi.2025.1685244

**Published:** 2025-11-25

**Authors:** Chenxi Pan, Enchun Zhao, Jingru Ren, Gaiyan Zhou, Yajie Wang, Ronggui Zhang, Yang Shen, Weiguo Liu, Jiu Chen

**Affiliations:** 1 Department of Anorectal, Huai'an TCM Hospital Affiliated to Nanjing University of Chinese Medicine, Huai’an, China; 2 Department of Neurology, The Affiliated Brain Hospital of Nanjing Medical University, Nanjing, China; 3 Department of Neurology, Tongji Hospital, School of Medicine, Tongji University, Shanghai, China; 4 Department of Neurology, Xiaogan Hospital Affiliated to Wuhan University of Science and Technology, The Central Hospital of Xiaogan, Xiaogan, China; 5 Department of Radiology, Nanjing Drum Tower Hospital, Affiliated Hospital of Medical School, Nanjing University, Nanjing, China; 6 Institute of Medical Imaging and Artificial Intelligence, Nanjing University, Nanjing, China; 7 Medical Imaging Center, Nanjing Drum Tower Hospital, Affiliated Hospital of Nanjing, Nanjing, China

**Keywords:** Parkinson’s disease, subjective cognitive decline, mild cognitive impairment, MRI, hippocampal subregion

## Abstract

**Background:**

Hippocampal subregion atrophy has been associated with cognitive decline in Parkinson’s disease (PD). Our study aimed to investigate the functional and structural alterations of the hippocampal subregion in newly diagnosed PD patients presenting with different cognitive states and to explore potential associations between these neuroanatomical alterations and cognitive performance.

**Methods:**

A total of four participant groups were recruited, including a *de novo* PD patient with mild cognitive impairment (PD-MCI, *n* = 44), a de novo PD patient with subjective cognitive decline (PD-SCD, *n* = 11), a de novo PD patient with normal cognition (PD-NC, *n* = 28), and a healthy control (HC, *n* = 19). The functional connectivity (FC) and gray matter (GM) volume alterations in the anterior, middle, and posterior regions of the left hippocampus were investigated. Moreover, we explored their relationship with cognition.

**Results:**

Compared to the PD-NC, the PD-SCD group revealed a significantly decreased FC between the left middle hippocampus (HIPm) and right postcentral gyrus (PoCG. R). Patients in the PD-MCI group had a decreased FC value not only between HIPm and PoCG. R but also between the left anterior hippocampus (HIPa) and PoCG. R. The GM volume in HIPa of PD-NC patients was significantly higher than that of PD-MCI patients. Furthermore, partial correlation analysis indicated that the FC in HIPa-PoCG. R and HIPm-PoCG. R was positively associated with executive functions. The elevated GM volume in HIPa was associated with better visuospatial and language functions.

**Conclusion:**

Dysfunction of left HIPm may contribute to initial executive decline in PD. The different subregions of the long-axis hippocampus could be targeted for early intervention strategies in PD with different cognitive states in the future.

## Introduction

Parkinson’s disease (PD) is one of the most common age-related brain disorders. Over 1.7 million people more than 55 years of age have been diagnosed with the condition in China (Zhang et al., 2005; Aarsland et al., 2017). Apart from its classical quartet of motor signs, PD results in a heterogeneous spectrum of non-motor symptoms (NMS), including cognitive impairment, which contributes significantly to the poor quality of life and institutionalization (Schapira et al., 2017; Lawson et al., 2014; Aarsland et al., 2000). A recent study demonstrates the full spectrum of cognitive impairment in PD, from subjective cognitive decline (PD-SCD) and mild cognitive impairment (PD-MCI) to dementia (PDD; Aarsland et al., 2021). A longitudinal follow-up study observed that 66.7% of PD-SCD patients reverted to normal cognition, while 33.3% developed PDD (Galtier et al., 2019). Moreover, 27.8% of PD-MCI patients reverted to normal cognition at the end of a 5-year population-based study (Pedersen et al., 2017). Therefore, patients at risk of cognitive decline can be identified earlier through the discovery of early biomarkers in PD patients with SCD or MCI, enabling timely clinical intervention and disease management.

Functional magnetic resonance imaging (MRI), which measures changes in blood flow, enables the study of functionally connected brain networks (Bonifacio and Zamboni, 2016) and has become a primary method for investigating the neural bases of cognitive deficits in Parkinson’s disease (Gorges et al., 2015; Peraza et al., 2017; Hajebrahimi et al., 2024). In particular, MRI evidence has identified the hippocampus as a notable predictor of cognitive disorder in PD (Delgado-Alvarado et al., 2016; Mak et al., 2015). This may be because the hippocampus is highly interconnected with other important brain structures (Schultz and Engelhardt, 2014; Rubin et al., 2014) and is extensively involved in various cognitive processes (Rubin et al., 2014; Simons and Spiers, 2003). Moreover, the hippocampus consists of several subfields with functional differentiation (Bai et al., 2019). In addition, Becker *et al.* found that considering the hippocampus as a homogeneous structure instead of a composite of subfields with distinct morphologies may have led to the inconsistent results of previous studies (Becker et al., 2021). Hippocampal subfield volumes can be more accurate biomarkers than global hippocampal volume for the early detection of dementia (La Joie et al., 2013). Importantly, previous studies with larger cohorts (Low et al., 2019) and advanced analytic approaches (Becker et al., 2021; La et al., 2019) indicated that changes in the hippocampal subfields were closely associated with cognitive decline in PD. Longitudinal studies demonstrated that structural changes in the hippocampus subfields in PD could cause cognitive dysfunction (Foo et al., 2017; Xu et al., 2020). However, previous studies mainly focused on the structural transformation of the hippocampal subregion. Thus, little is known about the functional and structural alterations of the hippocampal subregion among newly diagnosed PD patients across the cognitive spectrum.

This study investigated functional and structural alterations of the hippocampal subregion in a large, well-characterized cohort of non-demented patients with PD. It was hypothesized that PD-SCD and PD-MCI patients experience distinct changes in the hippocampal subregion as the earliest stage of worsening cognition. Furthermore, alterations in the hippocampal subregion would be associated with executive dysfunction because cognitive deficits observed in prodromal and newly diagnosed PD patients were predominantly executive dysfunction (Monastero et al., 2018; Weintraub et al., 2017).

## Materials and methods

### Subjects

This investigation was part of an ongoing longitudinal PD study. A total of 98 newly diagnosed PD patients and 21 healthy controls (HCs) were prospectively recruited from the Movement Disorders Unit of the Affiliated Brain Hospital of Nanjing Medical University from February 2019 to April 2021. A movement disorder specialist diagnosed patients with PD based on the UK Parkinson’s Disease Society Brain Bank Clinical Diagnostic Criteria (Gibb and Lees, 1988). HCs without any subjective cognitive complaint and MCI were recruited for comparison. The inclusion and exclusion criteria for PD and HC have been described in our previous study (Pan et al., 2022; Pan et al., 2021). In total, nine PD patients who refused or failed to complete an MRI scan were excluded.

The study was approved by the Medical Ethics Committee of the Affiliated Brain Hospital of Nanjing Medical University and completed in accordance with the Declaration of Helsinki. Written informed consent was obtained from all the participants.

### Clinical and neuropsychological assessment

All the subjects underwent a comprehensive evaluation, including clinical history, neurologic examination, and neuropsychological assessment, with two specialized neurologists assessing clinical characteristics. The motor symptoms were evaluated using the Unified Parkinson’s Disease Rating Scale Part III (UPDRS-III), while non-motor symptoms were assessed using the Non-Motor Symptoms Scale (NMSQ). The activities of daily living and disease stage were assessed using the UPDRS Part II (UPDRS-II) and the Hoehn and Yahr (H-Y) Scale, respectively. Global cognition was assessed using the Mini-Mental State Examination (MMSE) and the Montreal Cognitive Assessment (MoCA; Folstein et al., 1975; Nasreddine et al., 2005). In addition, depression and anxiety symptoms were evaluated with the Hamilton Anxiety Scale (HAMA) and the Hamilton Depression Rating Scale (HAMD).

All the subjects underwent a standard, comprehensive neuropsychological battery as described previously (Pan et al., 2021). The assessment was specifically designed to target cognitive impairment in a PD patient according to the guidelines of the Movement Disorder Society (MDS) Task Force (Litvan et al., 2012). A neuropsychological battery includes at least two tests in each of the five cognitive domains (i.e., attention and working memory, executive, memory, visuospatial, and language functions). Attention and working memory were measured using the Digit Span Test (DST), Trail Making Test A (TMT-A), and Stroop Color-Word Test (SCWT), while the executive functions were determined with the Trail Making Test B (TMT-B), Verbal Fluency Test (VFT), and Clock Drawing Test (CDT). Memory was assessed using the Auditory Verbal Learning Test (AVLT) and Logical Memory Test (LMT), while the visuospatial function was evaluated with Benton’s Judgment of Line Orientation Test (JLOT) and Hooper Visual Organization Test (HVOT). Language was assessed with the Boston Naming Test (BNT) and Wechsler Adult Intelligence Scale III (WAIS-III) Similarities Test. Subjects who failed to complete more than one test in each domain were excluded.

According to MDS level II criteria (Litvan et al., 2012), PD patients were classified as having MCI if two or more tests in the neuropsychological battery were impaired. A score ≥ 1.5 standard deviations (SDs) below the normative mean was considered impaired for a given test. The patients who did not meet the criteria for PD-MCI were classified as PD with normal objective cognitive performance. We classified PD-SCD as follows: (1) PD patients with normal objective cognitive performance; (2) the presence of subjective cognitive complaints, defined by the Cognitive Complaints Interview (CCI) score > 3 based on the patients’ and family members’ reports (Thomas-Antérion et al., 2006). The patients with normal objective cognitive performance and those who did not meet the criteria for SCD were considered PD patients with normal cognition (PD-NC).

For analysis purposes, the raw scores of each PD patient were transformed into z scores depending on the mean and SDs of the whole PD group. Then, the domain z-score was obtained by determining the average z-score of the variables in that domain.

### MRI procedure and image preprocessing

Images were acquired on a 3 T Verio Siemens scanner (Germany). As the scanning parameters have been detailed in our previous studies (Pan et al., 2022), they are not repeated here but are provided in the Supplementary materials and methods section. All MRI data preprocessing was carried out using Data Processing and Analysis for Brain Imaging (DPABI 4.3),^1^ which is based on the Statistical Parametric Mapping (SPM) program (SPM12),^2^ and implemented using MATLAB 2014b.^3^

The preprocessing of resting-state functional MRI (rs-fMRI) data is described in our previous study (Pan et al., 2022), which is as follows: (i) the first 10 volumes were removed for signal equilibrium, following which the images were realigned to the first volume to correct for the interscan head motion; (ii) structural and functional images were co-registered; (iii) motion artifacts (Friston 24-parameter), linear trend, and white matter (WM) and cerebral spinal fluid (CSF) signals were removed; (iv) images were normalized to the Montreal Neurological Institute (MNI) space using the Diffeomorphic Anatomical Registration Through Exponentiated Lie Algebra (DARTEL) toolbox and resampled to a voxel size of 3 mm × 3 mm × 3 mm; (v) and spatial smoothing was performed using a 6-mm full width-half-maximum Gaussian kernel and filtered through a 0.01–0.08 Hz bandpass filter.

The preprocessing of structural MRI data consisted of the following steps: (i) all T1-weighted images were initially subjected to spatial normalization to develop a study-specific template; (ii) T1-weighted of each subject were corrected for bias-field inhomogeneity and subjected to skull stripping and segmentation into gray matter (GM), WM, and CSF; (iii) the segmented GM, WM, and CSF were normalized to the MNI stereotactic space using the DARTEL toolbox, resulting in normalized GM images with a voxel size 1.5 × 1.5 × 1.5 mm^3^; (iv) the normalized GM images were modulated and smoothed with 6-mm full-width-half-maximum (FWHM) Gaussian. Finally, the GM volume map obtained from the subjects was included in further analyses.

### Image quality and motion control

Before data preprocessing, the raw MRI images were manually reviewed to exclude obvious artifacts and intracranial space-occupying lesions for quality assurance, resulting in the exclusion of one HC and one PD patient. Several approaches were employed to minimize potential head-motion bias, as a high level of head motion can significantly impact functional connectivity (FC; Van Dijk et al., 2012). First, during the MRI scan, all subjects lay with their head fixed in place using foam pads, and an 8-channel head coil was used to minimize head movement. Second, the subjects with excessive head motions (cumulative translation or rotation > 2.0 mm or 2.0°) were excluded. In total, five PD patients and one HC were excluded due to excessive head motion (*n* = 6). Third, the Friston 24-parameter model was utilized to regress out head motion effects from the realigned data. Finally, if the data showed mean framewise displacement (FD) > 0.5 mm (Brady et al., 2019), they were discarded, and no significant difference was observed in mean FD among groups (Table 1).

**Table 1:** Demographic and clinical characteristics of PD patients and healthy controls.

Variables	PD-MCI	PD-SCD		PD-NC	HC	*p*-value
	*n* = 44	*n* = 11		*n* = 28	*n* = 19	
Age (y)	59.89 ± 6.25	56.91 ± 6.27		56.50 ± 6.98	59.95 ± 5.74	0.099
Gender, male (%)	15 (34.1%)	8 (72.7%)		17(60.7%)	7(36.8%)	**0.033**
Education (y)	9.52 ± 3.18	10.46 ± 1.69		11.14 ± 3.23	10.76 ± 2.23	0.120
Disease duration (m)	15.00 ± 9.50	20.91 ± 11.74		25.00 ± 17.05	-	**0.022** ^**a**^
H-Y stage	1.64 ± 0.51	1.59 ± 0.30		1.48 ± 0.44	-	0.535
UPDRS ADL score	7.30 ± 3.63	8.73 ± 4.29		6.75 ± 3.12	-	0.301
UPDRS motor score	22.41 ± 10.42	17.82 ± 7.57		17.36 ± 7.82	0.42 ± 1.39	**0.000** ^**c, d, e**^
Motor phenotype						0.459
TD (%)	10 (22.7%)	3 (27.3%)		7 (25.0%)		
Indeterminate (%)	8 (18.2%)	2 (18.2%)		1 (3.6%)		
PIGD (%)	26 (59.1%)	6 (54.5%)		20 (71.4%)		
NMSQ	8.02 ± 5.03	10.18 ± 6.13		7.25 ± 3.82	1.53 ± 1.54	**0.000** ^**c, d, e**^
HAMA	7.11 ± 3.87	8.64 ± 10.47		5.14 ± 4.86	0.26 ± 0.93	**0.000** ^**c, d, e**^
HAMD	11.23 ± 6.01	12.73	± 11.64	7.68 ± 6.48	0.68 ± 1.89	**0.000** ^**c, d, e**^
MMSE	27.50 ± 2.14	28.18 ± 0.98		28.14 ± 1.76	28.16 ± 1.26	0.614
MOCA	21.61 ± 3.28	24.27 ± 3.80		25.39 ± 2.89	26.16 ± 2.36	**0.000** ^**a, b, c, d**^
CCI	3.98 ± 2.72	5.27 ± 1.49		1.75 ± 1.27	1.37 ± 0.29	**0.000** ^**a, b, c, d**^
Mean FD	0.09 ± 0.05	0.12 ± 0.08		0.09 ± 0.05	0.12 ± 0.08	0.201

The data are reported as mean ± SD or n (%). The results of *post-hoc* comparisons (Bonferroni or Games-Howell correction for one-way ANOVA and Bonferroni for Kruskal-Wallis H-test) are indicated as: ^a^Statistically significant difference between PD-MCI and PD-NC. ^b^Statistically significant difference between PD-SCD and PD-NC. ^c^Statistically significant difference between PD-MCI and HC. ^d^Statistically significant difference between PD-SCD and HC. ^e^Statistically significant difference between PD-NC and HC. The values in bold are statistically significant differences (*p* < 0.05). ADL, activities of daily living; CCI, Cognitive Complaints Interview; FD, framewise displacement; HAMA, Hamilton Anxiety Scale; HAMD, Hamilton Depression Rating Scale; HC, healthy control; H-Y, Hoehn and Yahr; m, month; MMSE, Mini-Mental State Examination; MOCA, Montreal Cognitive Assessment; NMSQ, Non-Motor Symptoms Scale; PD-MCI, Parkinson’s disease with mild cognitive impairment; PD-NC, Parkinson’s disease with normal cognition; PD-SCD, Parkinson’s disease with subjective cognitive decline; PIGD, postural instability gait difficulty; TD, tremor-dominant; UPDRS, Unified Parkinson’s Disease Rating Scale; y, year.

### Definition of left hippocampal subregions

Previous studies using advanced neuroimaging techniques have highlighted the functional difference between the hippocampus and its subregions (Poppenk and Moscovitch, 2011; Genon et al., 2021; Ushakov et al., 2016; Robinson et al., 2016; Chen et al., 2022). Studies have shown that the posterior hippocampus is critical for memory and cognitive processing, while the anterior hippocampus plays a greater role in other complex behaviors (Fanselow and Dong, 2010; Small et al., 2011). Furthermore, existing evidence suggests that the left hippocampus is more susceptible to pathological changes than the right (Small et al., 2011; Narr et al., 2004). Building on this, coactivation-based parcellation of task-fMRI data from the BrainMap database resulted in further segmentation of the left hippocampus into three distinct functional clusters: an anterior-most emotional cluster, a middle cognitive cluster, and a posterior-most perceptual cluster (Robinson et al., 2015). This classification system has also been applied to patients with depression and subjective cognitive decline to investigate the relationship between hippocampal subregional function and cognitive performance (Bai et al., 2019; Chen et al., 2020). Based on the foregoing parcellation, we adopted the three resulting subregions of the left hippocampus as our regions of interest (ROIs): the anterior subregion of the left hippocampus (HIPa), the middle subregion of the left hippocampus (HIPm), and the posterior subregion of the left hippocampus (HIPp; Bai et al., 2019; Robinson et al., 2015; Chen et al., 2020).

### Resting-state functional connectivity analysis

The resting-state functional connectivity (RSFC) was calculated using the DPABI software. For each subject, Pearson’s correlation coefficients were calculated between the average time course of each ROI and the voxel-wise time series across the rest of the brain within the group-specific whole-brain GM mask. The correlation coefficient was converted to a z-value using Fisher’s r-to-z transformation to improve normality. Thus, results were displayed using RSFC maps for each subject. The analysis of covariance (ANCOVA) was then applied to determine the difference in RSFC of each ROI among PD-SCD, PD-MCI, PD-NC, and HC groups, controlling for the effects of age, sex, education, and whole-brain GM volume. A significant cluster was detected when the two-tailed *p-*value < 0.05 and cluster size > 100 voxels. Based on the methodological implementation of the Permutation Analysis of Linear Models (PALM) package and the statistical properties of Threshold-free Cluster Enhancement (TFCE), the number of permutations was determined to be 1,000 (Winkler et al., 2016; Smith and Nichols, 2009). Subsequently, two-sample T-tests were conducted for *post-hoc* comparisons within the mask derived from ANCOVA, controlling for age, sex, education, and GM volume. A significant cluster threshold was set at a two-tailed TFCE with Family-wise Error (FWE) corrected *p*-value < 0.05 and cluster size > 20 voxels in our analyses (1,000 permutations in FWE evaluation).

### Voxel-based morphometry analysis

VBM analysis was applied to the structural MRI data after preprocessing. The GM volume maps of hippocampal subregions within the corresponding ROI were obtained using DPABI software for each individual. The mean GM volume was then calculated for the hippocampal subregion of each subject with in-house code running in MATLAB. Finally, the mean GM volume data were analyzed using the Statistical Package for the Social Sciences (SPSS) statistical software package (version 25.0). One-way analysis of variance (ANOVA) was used to compare the group differences among four groups, and Bonferroni correction was used for *post-hoc* comparisons. The result was deemed significant if the corrected *p*-value was < 0.05.

### Correlation analysis

Partial correlation analysis was performed in SPSS software to determine the association between hippocampal subregional alterations (both functional and structural) and cognitive domains in PD patients, while controlling for the effects of age, gender, years of education, and HAMD. The statistical significance was determined at *p*-value < 0.05 (two-tailed, Bonferroni-corrected).

### Statistical analysis

Demographic, neuropsychological, and clinical data were reported as the mean ± SD. The normality of clinical and demographic data distribution was checked using the Shapiro–Wilk test. One-way analysis of variance or Kruskal–Wallis test was used to compare continuous variables between HC and PD patient subtypes. Subsequently, the Bonferroni or Games-Howell *post-hoc* test was utilized after ANOVA, and the Bonferroni *post-hoc* test was used after the Kruskal–Wallis test, based on the normality of distribution and homogeneity of continuous variance. The chi-square test was utilized to compare the categorical variables. For the small amount of missing data (all < 5%) in the cognitive test, multiple imputation under the missing at random (MAR) assumption was performed. The imputation was performed using a Fully Conditional Specification (FCS) framework with a Markov Chain Monte Carlo (MCMC) algorithm, for which the number of iterations followed the default setting of the statistical software (SPSS). All the statistical analyses were conducted using SPSS software. Statistical significance was set at *p*-value < 0.05.

## Results

### Demographic and neurocognitive characteristics

Demographics and clinical characteristics of the sample, comprising 28 PD-NC, 11 PD-SCD, 44 PD-MCI, and 19 HCs, are summarized in Table 1. No significant differences in age, gender, years of education, H-Y score, UPDRS-II score, and MMSE were observed between any two groups (corrected *p* > 0.05). Compared to the HC group, the PD subgroups had higher NMSQ, HAMA, and HAMD scores (corrected *p* < 0.001). Patients with PD-SCD and PD-MCI had more cognitive complaints than the HC group and the PD-NC group (corrected *p* < 0.005). Patients with PD-MCI and PD-SCD had higher HAMD scores than PD-NC patients but without any significant differences (corrected *p* > 0.05).

Significant differences were observed among the four groups in all the neuropsychological tests (Table 2). In addition, the *post-hoc* test illustrated that the PD-MCI group had the worst performance in five cognitive domains compared to the HC group and the PD-NC group (corrected *p* < 0.05). Moreover, the PD-SCD group performed better in TMT-A, TMT-B, and HVOT than the PD-MCI group (corrected *p* < 0.05). However, there was no significant difference in any neuropsychological test among the PD-SCD, PD-NC, and HC groups (corrected *p* > 0.05).

**Table 2:** Cognitive performances of all participants.

Variables	PD-MCI	PD-SCD	PD-NC	HC	*p*-value
	*n* = 44	*n* = 11	*n* = 28	*n* = 19	
Attention/Working memory					
DST	11.45 ± 2.39	11.64 ± 2.16	12.75 ± 1.80	11.16 ± 1.86	**0.041**
DST-forward	7.16 ± 1.57	7.00 ± 1.41	8.00 ± 1.02	7.00 ± 1.56	**0.029**
DST-backward	4.30 ± 1.32	4.64 ± 1.03	4.71 ± 1.08	4.16 ± 0.96	0.108
TMT-A (s)	118.00 ± 55.75^#^	71.18 ± 20.38	74.11 ± 24.91	78.95 ± 13.54	**0.000** ^**a, b, c**^
SCWT-A-time	29.74 ± 9.14^#^	28.82 ± 8.94	25.54 ± 5.76	24.21 ± 4.64	**0.017** ^**c**^
SCWT-B-time	44.98 ± 18.52^#^	42.55 ± 11.43	35.41 ± 6.54^#^	38.26 ± 14.80	**0.029**
SCWT-C-time	79.58 ± 29.26^#^	83.73 ± 29.91	65.81 ± 13.49^#^	61.00 ± 9.60	**0.008** ^**c**^
SCWT-A-right	49.91 ± 0.37^#^	49.82 ± 0.41	49.96 ± 0.19	48.16 ± 5.36	0.318
SCWT-B-right	48.51 ± 3.03^#^	48.91 ± 2.17	49.74 ± 0.66^#^	49.47 ± 1.07	0.213
SCWT-C-right	46.77 ± 5.08^#^	47.45 ± 5.09	48.30 ± 2.87^#^	47.63 ± 2.17	0.204
Executive					
TMT-B (s)	238.56 ± 113.51^#^	152.18 ± 29.03	148.14 ± 31.3	153.42 ± 30.24	**0.000** ^**a, b, c**^
CDT	7.95 ± 2.57	9.27 ± 1.62	9.75 ± 0.84	9.95 ± 0.23	**0.000** ^**a, c**^
VFT	17.07 ± 5.74	17.82 ± 3.95	20.64 ± 4.19	18.05 ± 2.97	**0.001** ^**a**^
Memory					
AVLT-delayed recall	4.77 ± 2.49	6.27 ± 1.42	6.21 ± 2.35	6.79 ± 2.82	**0.011** ^**c**^
AVLT-recognition	21.43 ± 2.48	22.55 ± 1.44	22.36 ± 1.47	23.05 ± 1.08	**0.031** ^**c**^
LMT-delayed recall	4.88 ± 2.54	6.05 ± 2.80	6.66 ± 1.69	6.42 ± 2.47	**0.010** ^**a**^
Visuospatial function					
JLOT	22.69 ± 3.48	25.55 ± 2.46	25.04 ± 2.16	24.76 ± 2.48	**0.001** ^**a, b**^
HVOT	12.18 ± 4.54	17.36 ± 4.11	17.98 ± 3.37	16.61 ± 4.11	**0.000** ^**a, b, c**^
Language					
similarities	14.14 ± 4.03	17.36 ± 4.93	19.07 ± 3.19	16.16 ± 4.30	**0.000** ^**a**^
BNT	22.20 ± 4.29	25.09 ± 2.02	25.82 ± 2.37	24.95 ± 3.21	**0.001** ^**a, c**^

The raw scores of neuropsychological tests are presented as mean ± SD. The results of *post-hoc* comparisons (Bonferroni or Games-Howell correction for one-way ANOVA, and Bonferroni for Kruskal-Wallis H-test) are indicated as follows: ^a^Statistically significant difference between PD-MCI and PD-NC. ^b^Statistically significant difference between PD-MCI and PD-SCD. ^c^Statistically significant difference between PD-MCI and HC. The values in bold are statistically significant differences (*p* < 0.05). # One participant did not finish the test. AVLT, Auditory Verbal Learning Test; BNT, Boston Naming Test; CDT, Clock Drawing Test; DST, Digit Span Backward Test; HC, healthy control; HVOT, Hooper Visual Organization Test; JLOT, Benton’s Judgment of Line Orientation Test; LMT, Logical Memory Test; PD-MCI, Parkinson’s disease with mild cognitive impairment; PD-NC, Parkinson’s disease with normal cognition; PD-SCD, Parkinson’s disease with subjective cognitive decline; s, second; SCWT, Stroop Color-Word Test; TMT-A, Trail Making Test A; TMT-B, Trail Making Test B; VFT, Verbal Fluency Test.

### VBM

**Figure 1:**
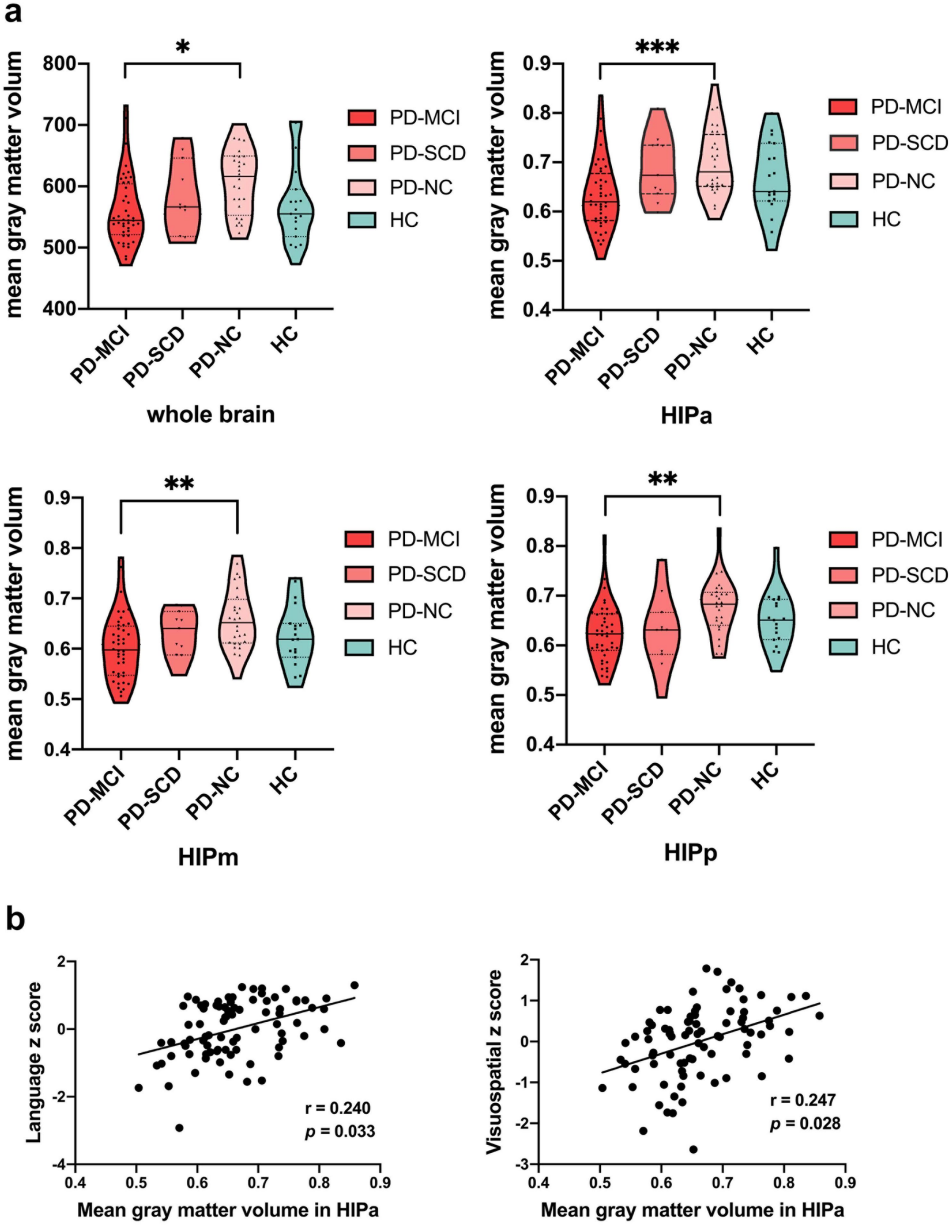
The results of structural alterations in hippocampal subregions. **(a)** Comparisons of gray matter volume in the whole-brain and hippocampus subregions among Parkinson’s disease subgroups and healthy controls were plotted with violin plots (*p < 0.05; **p < 0.005; and ***p = 0.001). **(b)** Correlations between the gray matter volume in HIPa and cognitive domains. HC, healthy control; HIPa, anterior of left hippocampus; HIPm, middle of left hippocampus; HIPp, posterior of left hippocampus; PD-MCI, Parkinson’s disease with mild cognitive impairment; PD-NC, Parkinson’s disease with normal cognition; PD-SCD, Parkinson’s disease with subjective cognitive decline.

The PD-NC group exhibited elevated GM volume in HIPa, HIPm, HIPp, and whole-brain than the PD-MCI group (corrected *p* < 0.05; Figure 1a). Although the GM volume in HIPa of PD-SCD patients was higher than that of PD-MCI patients, the difference was statistically insignificant. No significant differences in GM volume within the hippocampal subregions were observed between any two of the PD-SCD, PD-NC, and HC groups. Moreover, the normalized whole-brain GM volume was used as a covariate in FC analysis because it varied between PD subgroups. As described in Figure 1b, increased GM volume in HIPa was related to better visuospatial (*r* = 0.247, *p* = 0.028) and language (*r* = 0.240, *p* = 0.033) functions.

### RSFC

**Figure 2:**
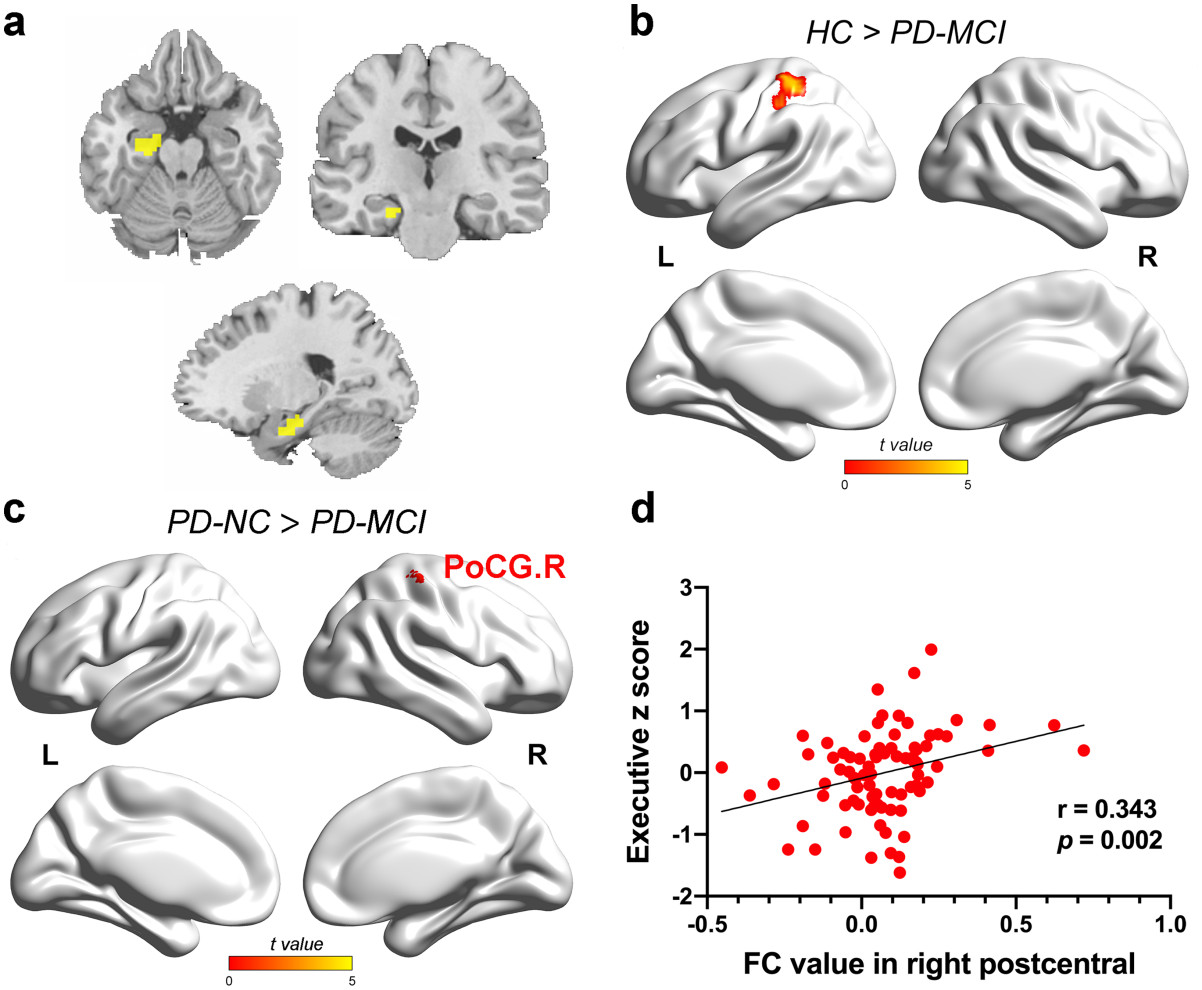
The results of FC alteration in the HIPa. **(a)** The coronal, axial, and sagittal views of HIPa. **(b)** The PD-MCI patients indicated decreased FC between HIPa and left POCG than HC (TFCE-FWE corrected *p* < 0.05, cluster size > 20 voxels). **(c)** The PD-MCI patients depicted reduced FC between HIPa and right POCG than PD-NC patients (TFCE-FWE corrected *p* < 0.05, cluster size > 20 voxels). **(d)** A significant relationship was observed between decreased FC in HIPa-POCG. R and executive functions (Bonferroni-corrected *p* < 0.05, two-tailed). The color bar encodes the uncorrected t-values for voxels within significant clusters (TFCE-FWE correction). FC, functional connectivity; HC, healthy control; HIPa, anterior of left hippocampus; PD-MCI, Parkinson disease with mild cognitive impairment; PD-NC, PD patients with normal cognition; PD-SCD, PD patients with subjective cognitive decline; POCG, postcentral gyrus; R right.

ANCOVA showed significantly altered FC across the four groups for the HIPa as ROI (Figure 2a), including left calcarine fissure and surrounding cortex (CAL), left inferior occipital gyrus (IOG), left superior occipital gyrus (SOG), bilateral middle occipital gyrus (MOG), bilateral superior temporal gyrus (STG), bilateral postcentral gyrus (PoCG), bilateral precentral gyrus (PreCG), bilateral paracentral lobule (PCL), and left superior frontal gyrus, dorsolateral (SFGdor). Between-group comparisons revealed that the PD-MCI group showed significantly reduced FC in left PoCG (TFCE-FWE corrected *p* < 0.05; Figure 2b; Supplementary Table 1) and right PoCG (TFCE-FWE corrected *p* < 0.05; Figure 2c; Supplementary Table 1) compared to the HC group and the PD-NC group.

**Figure 3:**
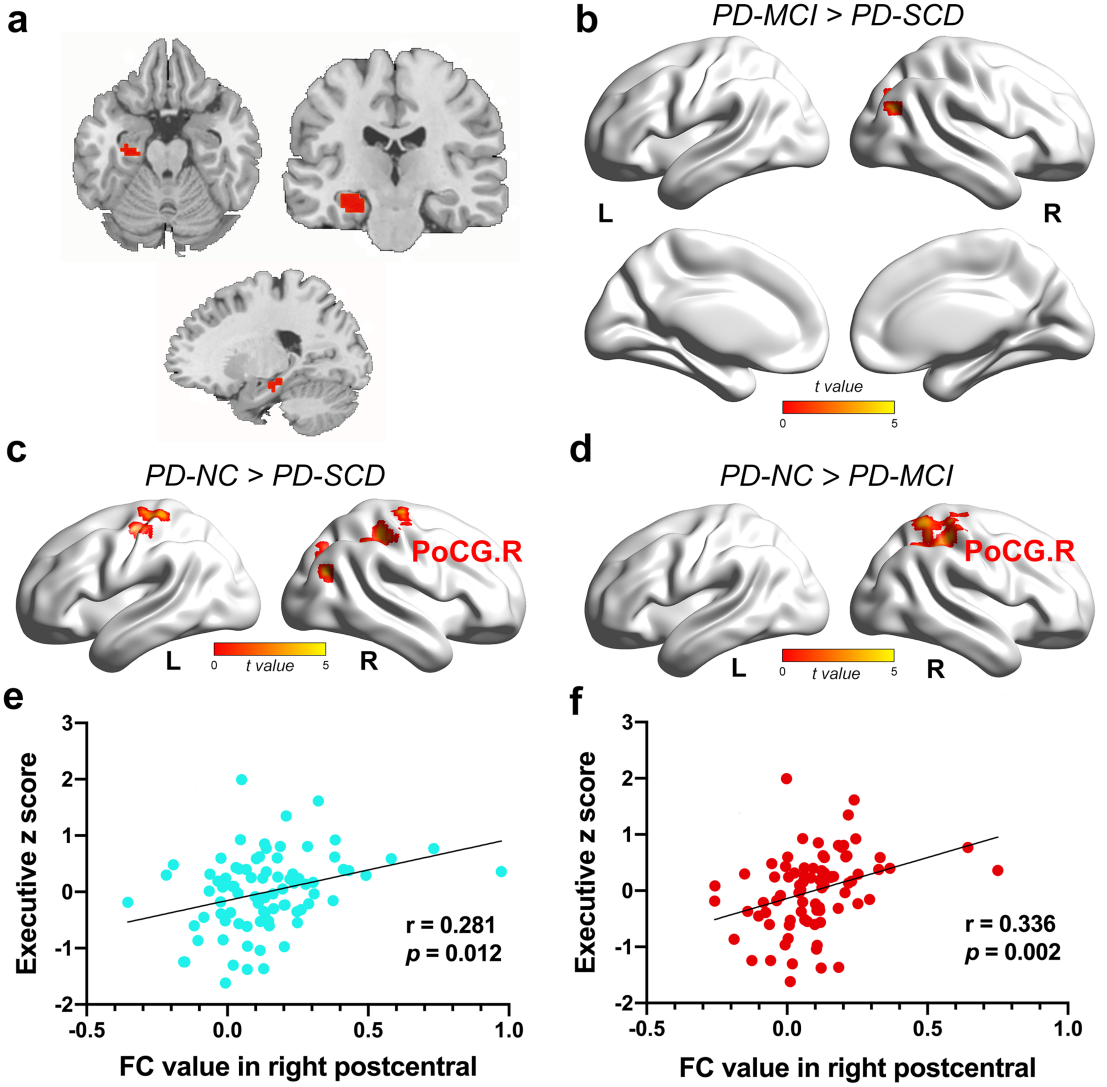
The results of FC alteration in the HIPm. **(a)** The coronal, axial, and sagittal views of HIPm. **(b)** The PD-MCI patients indicated increased FC between HIPm and right MOG than PD-SCD patients (TFCE-FWE corrected *p* < 0.05, cluster size > 20 voxels). **(c)** The PD-SCD patients revealed reduced FC between HIPm and bilateral PoCG, right MOG, right SOG, and bilateral PreCG than PD-NC patients (TFCE-FWE corrected *p* < 0.05, cluster size > 20 voxels). **(d)** The PD-MCI patients demonstrated decreased FC between HIPm and right PoCG, SPG, and PreCG than PD-NC patients (TFCE-FWE corrected *p* < 0.05, cluster size > 20 voxels). **(e)** FC values in HIPm-POCG. R connection (PD-SCD < PD-NC group) were positively correlated to executive functions (uncorrected *p* < 0.05, two-tailed). **(f)** FC values in HIPm-POCG. R connection (PD-MCI < PD-NC group) were positively correlated with executive functions (Bonferroni-corrected *p* < 0.05, two-tailed). The color bar encodes the uncorrected t-values for voxels within significant clusters (TFCE-FWE correction). FC, functional connectivity; HIPm, middle of left hippocampus; MOG, middle occipital gyrus; POCG, postcentral gyrus; PreCG, precentral gyrus; R right; SOG, superior occipital gyrus; SPG, superior parietal gyrus.

Furthermore, ANCOVA showed significantly altered FC among the four groups for HIPm as ROI (Figure 3a), including right parahippocampal gyrus (PHG), bilateral PoCG, bilateral PreCG, right superior parietal gyrus (SPG), right superior occipital gyrus (SOG), and right MOG. The findings between two groups showed the following: (1) the PD-SCD patients indicated significantly decreased FC in bilateral PoCG, right MOG, right SOG, and bilateral PreCG than the PD-NC patients (TFCE-FWE corrected *p* < 0.05; Figure 3c; Supplementary Table 1); (2) the PD-MCI group showed significantly reduced FC in right PoCG, right SPG, and PreCG than the PD-NC group (TFCE-FWE corrected *p* < 0.05; Figure 3d; Supplementary Table 1); (3) the PD-MCI group illustrated significantly elevated FC in right MOG than the PD-SCD group (TFCE-FWE corrected *p* < 0.05; Figure 3b; Supplementary Table 1).

For the HIPp as ROI, ANCOVA analysis revealed an altered FC between HIPp and left lingual gyrus (LING). The PD-SCD group revealed significantly decreased FC in left LING than the HC group and the PD-NC group (TFCE-FWE corrected *p* < 0.05; Supplementary Table 1; Supplementary Figure 1).

In the full PD sample, FC between HIPa and right PoCG was positively correlated with executive function (HIPa-POCG. R; *r* = 0.343, *p* = 0.002, Bonferroni-corrected; Figure 2d). Similarly, FC between HIPm and right PoCG (HIPm-PoCG. R) was positively associated with executive functions (*r* = 0.281, *p* = 0.012, Figure 3e; *r* = 0.336, *p* = 0.002, Figure 3f; Bonferroni-corrected). These results were obtained after controlling for the effects of age, sex, years of education, and HAMD score.

## Discussion

This study, investigating functional and structural alterations of hippocampal subregions of the left hippocampus in initial cognitive decline PD patients, showed four major findings: (1) the FC between HIPm-PoCG. R decreased in PD-SCD patients, despite objective cognition and hippocampus structure of the PD-SCD patients being in the normal range; (2) except for the decreased FC in HIPm-PoCG. R, the PD-MCI indicated equally reduced FC in HIPa-PoCG. R. Furthermore, altered FC in HIPa-PoCG. R and HIPm-PoCG. R was related to executive function in PD; (3) the PD-NC patients showed increased GM volume in each subregion of hippocampus compared with the PD-MCI patients; and (4) the GM volume of HIPa was associated with visuospatial and language performance in PD.

A pathophysiological framework supports the view that a specific disease is associated with a specific hippocampal subregion (Small et al., 2011). Previous studies indicated that the anterior-to-posterior subregions of the hippocampus were associated with behavioral domains and neurocognitive processes (Bai et al., 2019; Poppenk and Moscovitch, 2011). Robinson et al. demonstrated that the anterior-most cluster is involved in emotional processing and that the middle cluster is involved in cognitive processing, with a data-driven approach (Robinson et al., 2015). However, Genon et al. hold the view that the anterior hippocampus has relative specificity in emotion processing but a more widespread cognition correlation (Genon et al., 2021). Moreover, neuroimaging studies suggested that the anterior hippocampus is generally affected in the early stages of psychosis (Kalmady et al., 2017; McHugo et al., 2018). We speculated that altered FC in HIPa and HIPm may reflect early cognitive dysfunction in PD patients, depending on the above evidence and our results.

Our study found that PD-SCD patients exhibit reduced FC between the HIPm and PoCG. R compared to PD-NC patients. Changes in PoCG have been widely reported in PD (Filippi et al., 2020; Zhang et al., 2018). Kim et al. observed that the nodal degree of the right postcentral gyrus is associated with cognitive function and indicated that the reduction in neural collaboration of the postcentral gyrus can lead to cognitive decline in the elderly with SCD (Kim et al., 2019). Notably, we observed a decreased FC in HIPm-PoCG. R related to poor executive performance in further analysis. Executive function is a commonly affected cognitive domain in newly diagnosed and prodromal PD (Monastero et al., 2018; Fengler et al., 2017), and the decline in executive function was related to the dysfunction of locus coeruleus (LC) neurons (Li et al., 2019). The hippocampus is a terminal field of LC, vital for cognitive behaviors (Borodovitsyna et al., 2017). Moreover, previous research identified that cortical thinning of right PoCG is associated with poorer executive performance in PD patients (Zhang et al., 2018). Based on the convergence of prior evidence and our current data, we speculate that the altered FC in the HIPm-PoCG. R circuit represents a characteristic functional alteration in PD-SCD patients—one that may contribute to executive decline and can precede the onset of obvious structural atrophy. In addition, we observed altered FC in the HIPa-PoCG. R circuit, along with the previously noted HIPm-PoCG. R circuit, in PD-MCI patients, with both alterations relating to executive performance. It has been suggested previously that any primary lesion or dysfunction in a subregion affects neighboring subregions over time due to the connection within the hippocampal circuit (Small et al., 2011). Based on our findings, we propose that dysfunction in HIPm-PoCG. R is a plausible neural substrate for the executive dysfunction characteristic of PD-MCI. In summary, our functional-level findings reveal a progressive pattern of hippocampal functional connectivity disruption from PD-SCD to PD-MCI, which follows a posterior-to-anterior gradient and corresponds with the trajectory of cognitive decline. It should be noted, however, that this was a pilot exploratory study with a limited sample size, especially in the PD-SCD subgroup, which may have reduced statistical power and increased the risk of type II errors. Therefore, the findings warrant cautious interpretation and await validation from future studies with larger cohorts.

Voxel-based morphometry analysis demonstrated that, at the structural level, the PD-NC group exhibited greater GM volume in key hippocampal subfields (HIPa, HIPm, and HIPp) than the PD-MCI group—a finding directly in line with previous studies associating hippocampal preservation with superior cognitive outcomes in PD (Low et al., 2019). A longitudinal MRI study reported an initial increase in cortical thickness in cognitively stable PD patients, characterizing it as an early phase phenomenon that follows an inverted U-shape function before the eventual dominance of atrophic processes (Filippi et al., 2020). Moreover, this inverted U-shape function is not unique to PD but has also been observed in Alzheimer’s Disease (AD), suggesting a potential shared mechanism in neurodegenerative processes (Sala-Llonch et al., 2015). Evidence from animal models demonstrates that the inflammatory response triggered by Aβ load directly drives the observed volume increase measured by MRI (Maheswaran et al., 2009). Furthermore, the study revealed a strong correlation between greater GM volume in the left (Lehrner et al., 2014; Baschi et al., 2018) anterior hippocampus and better visuospatial and language performance, suggesting a link between the structural integrity of this region and cognitive function. Previous research has established that specific substructures within the anterior hippocampus, primarily composed of the Cornu Ammonis 1 (CA1) and subiculum, critically support not only episodic memory but also imagination and visual scene perception (Zeidman and Maguire, 2016). Low et al. reported that in PD, the CA1 and subiculum were associated with impairments in language and executive function, respectively (Low et al., 2019). Furthermore, a previous PET imaging study identified higher relative [(Bai et al., 2019)F-FEOBV] uptake in the hippocampus, indicating a cognitive compensatory mechanism only in cognitively normal PD patients (Legault-Denis et al., 2021). Thus, the observed abnormalities in PD-NC patients were interpreted as a thicker cortex, which manifests early in PD patients, prior to atrophy, and may be a result of a cognitive compensatory mechanism. However, within the framework of longitudinal studies, whether this “increase” reflects a protective brain reserve mechanism or, conversely, a pathological process in PD patients with normal cognition remains to be determined.

Several limitations in the present study need to be considered. First, the PD-SCD subgroup had a relatively small sample size (*n* = 11), which may lead to insufficient statistical power and an increased risk of type II errors. This limitation stems directly from the low prevalence of PD-SCD within the newly diagnosed PD cohort. In the past, few researchers paid close attention to subjective cognition in PD. Moreover, the SCD prevalence ranges from 16.3 to 27.3% due to discrepancies in the study population, SCD definitions, and cognitive tests used.

However, a longitudinal study observed that only 10 (13.2%) of 76 *de novo* untreated PD patients met the current SCD criteria (subjective but not objective impairment), (Erro et al., 2014) which is consistent with our finding (11 in 98). Second, the current research was a cross-sectional study that was unable to verify FC changes in the HIP subregion as a biomarker for early cognitive decline in PD. Third, the generalizability of our findings was limited by the sole assessment of left hippocampal subregions. The conclusions would be strengthened by future bilateral analyses. Finally, disease duration was not used as a covariate in the RSFC analysis, and no covariates were included in the structural analysis. Future longitudinal studies with large, clinically matched cohorts should explore the dynamic alteration in FC patterns of the bilateral hippocampal subregion and determine whether these patterns can predict executive dysfunction in patients with early PD.

This is the first study, with a relatively large sample, exploring functional and structural changes in left hippocampal subregions of PD patients across the cognitive spectrum, ranging from normal cognition and SCD to MCI. The study illustrated that the functional pattern and structure of left hippocampal subregions differed between PD-SCD and PD-MCI patients. Altered functional patterns of left hippocampal subregions were associated with early executive decline in PD. Hence, the results revealed that the abnormal FC pattern of the left hippocampal subregion could be a promising imaging biomarker for cognitive decline in early PD. Thus, it needs to be verified through future longitudinal studies. Moreover, our findings could be highly informative for early interventions for cognitive decline in PD.

## Data Availability

The raw data supporting the conclusions of this article will be made available by the authors, without undue reservation.

## Footnotes

^1^http://rfmri.org/dpabi

^2^ http://www.fifil.ion.ucl.ac.uk/spm

^3^ http://www.mathworks.com/products/matlab
