# Supplementary material for: Multimodal MRI reveals distinct hippocampal subregional alterations in de novo Parkinson’s disease across the cognitive spectrum
Source: Front Aging Neurosci. 2025 Nov 25;17:1685244. doi: 10.3389/fnagi.2025.1685244 (PMC12685818; doi:10.3389/fnagi.2025.1685244)
Supplement: Supplementary file 1 [file Data_Sheet_1.docx]

***Supplementary material***

**Multimodal MRI reveals distinct hippocampal subregional alterations in de novo Parkinson’s disease across the cognitive spectrum**

Chenxi Pan, Enchun Zhao, Jingru Ren, Gaiyan Zhou, Yajie Wang, Ronggui Zhang, Yang Shen, Weiguo Liu*, Jiu Chen*****

*Correspondence to: Weiguo Liu and Jiu Chen

I. Supplementary Material and Methods

II. Supplementary Table 1

III. Supplementary Figure 1

*I. Supplementary Material and Methods*

MRI procedure

Images were acquired on a 3T Verio Siemens scanner. Structural 3D T1-weighted images were acquired by spoiled gradent echo (SPGR) with the following parameters: repetition time (TR) = 2530 ms, echo time (TE) = 3.34 ms, flip angle (FA) = 7 degrees, number of slices = 128, slice thickness = 1.33 mm, matrix = 256 × 192, field of view FOV = 256 mm × 256 mm, voxel size = 1×1×1 mm^3^. Resting state functional images were acquired using a gradient-recalled echo-planar (GRE-EPI) imaging pulse sequence with TR = 2000 ms, TE = 30 ms, FA = 90 degrees, number of slices = 31, slice thickness = 3.5 mm, matrix = 64× 64, FOV = 220 mm × 220 mm, number of total volumes = 240. During functional MRI, subjects were asked to lie quietly with their eyes closed, and remain awake but without thinking anything.

*II. Supplementary Table 1*

Supplementary Table 1. The region showing significant differences between two groups in seed-based functional connectivity analysis.

Abbreviations: HC, healthy control; HIPa, anterior of left hippocampus; HIPm, middle of left hippocampus; HIPp, posterior of left hippocampus; L, left; PD-MCI, Parkinson disease with mild cognitive impairment; PD-NC, Parkinson disease with normal cognition; PD-SCD, Parkinson disease with subjective cognitive decline; R, right.

|  | Brain regions (aal) | MNI coordinate  (x, y, z) | Cluster number (voxels) | Peak t value |
| --- | --- | --- | --- | --- |
| **Seed of HIPa** | |  |  |  |
| PD-MCI > HC | |  |  |  |
|  | L postcentral gyrus | -39, -27, 45 | 23 | - 3.647 |
| PD-MCI > PD-NC | |  |  |  |
|  | R postcentral gyrus | 42, -33, 66 | 72 | - 3.977 |
| **Seed of HIPm** | |  |  |  |
| PD-SCD > NC | |  |  |  |
|  | R middle occipital gyrus/ R superior occipital gyrus | 33, -69, 30 | 161 | - 3.996 |
|  | R postcentral gyrus | 39, -33, 51 | 52 | - 3.467 |
|  | L postcentral gyrus | -42, -21, 51 | 22 | - 3.372 |
|  | L precentral gyrus | -33, -24, 69 | 64 | - 3.789 |
|  | R precentral gyrus | 30, -24, 72 | 32 | - 3.95 |
| PD-MCI > PD-NC | |  |  |  |
|  | R postcentral gyrus /R superior parietal gyrus/ R precentral gyrus | 39, -36, 66 | 306 | - 4.114 |
| PD-MCI > PD-SCD | |  |  |  |
|  | R middle occipital gyrus | 33, -69, 30 | 104 | 4.125 |
| **Seed of HIPp** | |  |  |  |
| PD-SCD > HC | |  |  |  |
|  | L lingual gyrus | -6, -87, -15 | 130 | - 4.704 |
| PD-SCD > PD-NC | |  |  |  |
|  | L lingual gyrus | -33, -90, -21 | 85 | - 3.504 |

III. Supplementary Figure 1


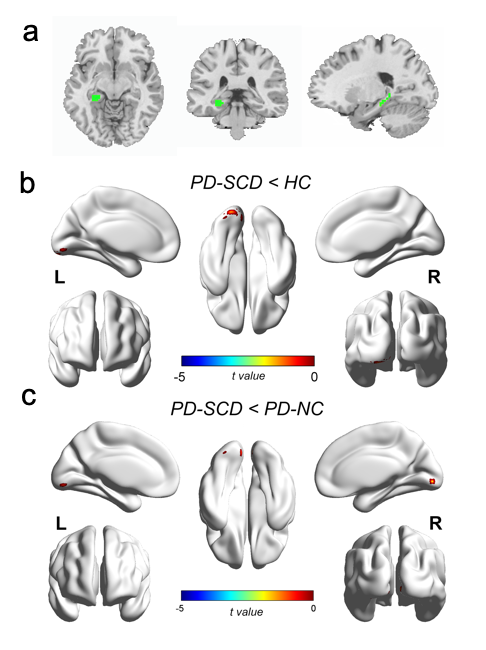


Supplementary Figure 1. Results of FC alteration in the HIPp. (a) The coronal, axial, and sagittal views of HIPp. (b) The PD-SCD patients show decreased FC between HIPp and left lingual gyrus than HCs (TFCE-FWE corrected p < 0.05, cluster size > 20 voxels). (c) The PD-SCD patients show decreased FC between HIPp and left lingual gyrus than PD-NC patients (TFCE-FWE corrected p < 0.05, cluster size > 20 voxels). The color bar encodes the uncorrected t-values for voxels within significant clusters.

Abbreviations: FC, functional connectivity; HCs, healthy controls; HIPp, posterior of left hippocampus; PD-NC, Parkinson disease with normal cognition; PD-SCD, Parkinson disease with subjective cognitive decline.
